# A Rare Case of Osmotic Demyelination Syndrome in Advanced Pancreatic Adenocarcinoma Without Hyponatremia

**DOI:** 10.7759/cureus.80406

**Published:** 2025-03-11

**Authors:** Konstantinos Baronos, Muhammad Memon

**Affiliations:** 1 Medicine, University Hospital Leicester, Leicester, GBR; 2 Acute Internal Medicine, University Hospital Leicester, Leicester, GBR

**Keywords:** central pontine myelinolysis, central pontine myelinolysis (cpm), metastatic pancreatic adenocarcinoma, metastatic pancreatic mass, osmotic demyelination syndrome (ods), pontine osmotic demyelination syndrome

## Abstract

Osmotic demyelination syndrome (ODS) is a rare neurological disease involving myelin loss in the central nervous system and is traditionally linked with abrupt correction of hyponatremia. In the absence of hyponatremia, ODS can occur if there are other risk factors such as malignancy, malnutrition, and metabolic disturbances. We describe a 52-year-old male with advanced pancreatic adenocarcinoma and liver metastases who presented with confusion, falls, bilateral leg oedema, hyperglycemia and hypokalemia. Initial imaging was negative for intracranial pathology but positive for pancreatic and hepatic malignancy. The patient's condition deteriorated despite intravenous insulin and potassium, and a brain MRI was positive for central pontine ODS without evidence of metastatic disease. Sodium levels were within normal limits on hospitalisation. A multi-disciplinary team deemed the malignancy inoperable, and the patient was transferred to palliative care and died three weeks post-discharge. This case illustrates the multifactorial aetiology of ODS in cancer patients, with a contribution from malignancy-related nutrient deficiencies, hypokalemia, and hyperglycemia despite normal sodium. Current literature identifies oligodendrocyte susceptibility to energy deficiency in cancer as a predisposition to ODS. This report highlights the significance of heightened awareness of ODS in malignancy, even in the absence of hyponatremia, and calls for further research into its pathophysiological basis as well as management.

## Introduction

Osmotic demyelination syndrome (ODS) is a rare neurological disorder with an incidence of 0.06% of all medical hospital admissions, characterised by the destruction of myelin in the central nervous system. It encompasses both central pontine myelinolysis (CPM), primarily affecting the pons, and extrapontine myelinolysis (EPM), which involves other brain regions. Adam et al. were the first to describe CPM in 1959 in patients with alcoholism and malnutrition [1]. The clinical manifestations vary but often involve encephalopathy or seizures, dysphagia, dysarthria, oculomotor dysfunction, and quadriparesis and may even become locked-in. ODS is most commonly associated with rapid correction of hyponatraemia, though other triggers such as hypoalbuminemia, hypokalemia, diabetes, liver transplantation, chronic alcoholism, infection and haematological malignancies have been implicated [2]. Malignancies often induce nutrient deficiencies that impair the energy metabolism of oligodendrocytes, essential for myelin repair, thereby predisposing patients to the development of ODS [3].

We present a rare case, which, to our knowledge, is the first reported instance of advanced pancreatic adenocarcinoma with osmotic demyelination syndrome and normal sodium levels.

## Case presentation

A 52-year-old, white, Caucasian male with a background of type 2 diabetes mellitus and hypertension presented to the Emergency Department with multiple recent falls, non-productive cough, bilateral leg swelling and confusion. His wife reported a noticeable change in his behaviour and cognition over the prior weeks. On physical examination, the patient was alert, oriented, Glasgow Coma Scale (GCS) 14/15, speaking in full sentences, had a small wound on the forehead from a recent fall, unremarkable respiratory, cardiac and abdominal examination and grade 4 bilateral pitting oedema.

Initial patient workup included routine blood tests which showed hypokalemia, hypoalbuminemia, hyperglycemia and a normal sodium level (Table 1). A chest X-ray was requested to investigate the presentation of the cough the patient had, which showed no evidence of effusion, consolidation or focal lesions. A computed tomography (CT) head was performed following multiple unwitnessed falls and evidence of facial injury, revealing no acute intracranial findings of bleed or lesions. Respiratory swabs taken on admission tested negative for Influenza and COVID-19 infection.

**Table 1:** The table summarises key blood test results obtained on Days 1, 5, and 10 of the patient’s hospital stay, alongside the reference range and units

Blood test	Day 1	Day 5	Day 10	Reference Range	Units
White Cell Count	6.8	7.6	6.6	4.0 - 11.0	x10^9/L
Haemoglobin	130	130	129	130 - 180	g/L
Haematocrit	0.364	0.372	0.375	0.400 - 0.540	L/L
Mean Cell Volume	86	89	88	80.0 - 99.0	fL
Platelet Count	93	72	89	140 - 400	x10^9/L
eGFR	>90	>90	>90		mL/min/1.73m2
Sodium	135	138	141	133 - 146	mmol/L
Potassium	2.5	3	2.9	3.5 - 5.3	mmol/L
Urea	4.6	4.2	4.2	2.5 - 7.8	mmol/L
Creatinine	76	67	65	60 - 120	umol/L
Alanine Transaminase	47	74	65	4 - 36	U/L
Total Bilirubin	17	16	16	0 - 21.0	umol/L
C-Reactive Protein	15	<5	<5	0 - 10	mg/L
Total Protein	54	53	55	57 - 82	g/L
Albumin	33	34	33	35 - 50	g/L
Serum Magnesium	1.02	0.87	0.88	0.7 - 1.0	mmol/L
Random Glucose Levels	21.1	18.7	14.3	5.6 - 6.9	mmol/L
Serum Osmolality	296	299	301	285 - 295	mOsm/kg
Bicarbonate	29.5			21.0 - 29.0	mmol/L

**Figure 1:**
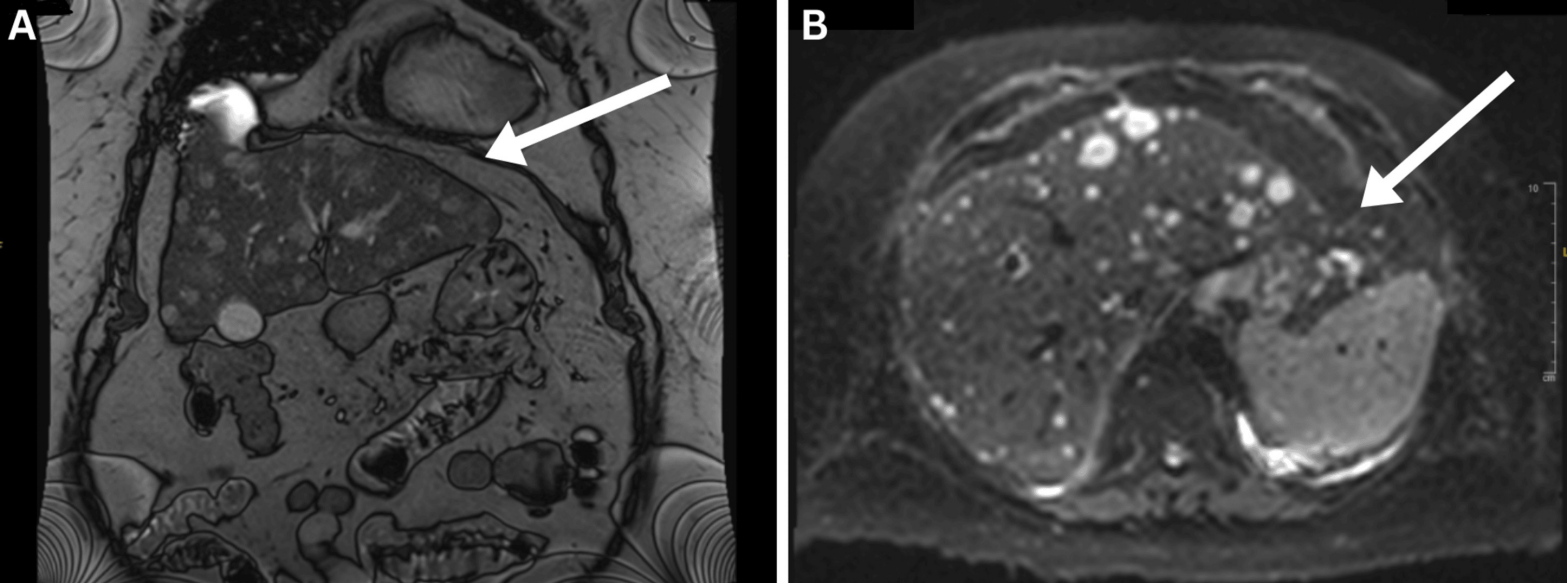
MRI of the liver and pancreas Multiple, innumerable, bilobar hepatic hypovascular metastases seen, with the index lesion in segment 8/5 measuring up to 25 x 21 mm (Image A). Ill-defined pancreatic distal body/tail intermediate T2 signal lesion measuring 5 x 6 cm seen replacing the fatty interdigitation close to the splenic hilum (Image B).

Given the patient's bilateral pitting oedema, an ultrasound of the urinary tract was requested to rule out renal pathology. The findings were unremarkable for renal or urinary pathology but incidentally identified multiple heterogeneous liver lesions highly suspicious for metastases, necessitating further cross-sectional imaging. Subsequent CT imaging of the thorax, abdomen and pelvis demonstrated a 3.5 cm mass in the pancreatic tail, suggestive of a primary malignancy, with multiple hypoenhancing hepatic lesions consistent with metastatic disease. Splenomegaly and unexplained portal hypertension were noted, along with patchy ground-glass opacities in both upper lung lobes, raising suspicion for viral pneumonitis. To further characterise the liver lesions, magnetic resonance imaging (MRI) of the liver and pancreas was performed on confirmed multiple bilobar hepatic metastases and a pancreatic mass (5 x 8 cm) in the distal body/tail, replacing fatty interdigitation near the splenic hilum. Mild restricted diffusion and suboptimal contrast enhancement were observed, supporting the suspicion of malignancy (Figure 1). Further laboratory testing showed cancer antigen (CA) 19.9 positivity with a value of 1388 U/mL and carcinoembryonic antigen (CEA) with a value of 42 ng/mL.

**Figure 2:**
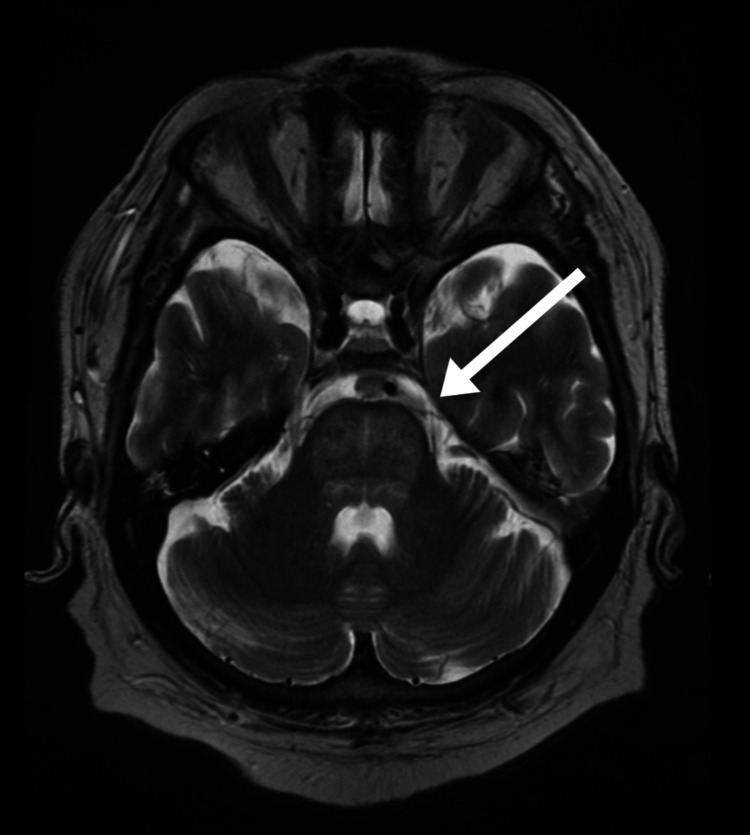
MRI brain showing central pontine hyperintensity on T2/FLAIR FLAIR: fluid-attenuated inversion recovery

While the hypokalemia was being pharmacologically corrected with intravenous and oral potassium supplementation, and the hyperglycemia managed by setting up a variable rate insulin infusion, the patient remained bed-bound, increasingly confused, and becoming less verbally expressive. This necessitated further head scans. MRI of the brain revealed a central pontine hyperintensity on T2/fluid-attenuated inversion recovery (FLAIR) sequences, consistent with ODS (Figure 2). No evidence of metastatic disease was identified in the brain. A serum neuronal antibody screen was performed, including antibodies for Purkinje cells, neuronal nuclei (Hu and Ri), anti-CV2, anti-amphiphysin and anti-Ma2, which all came back negative.

A multidisciplinary team (MDT) meeting of consultants from various specialities, including oncology, surgeons, radiologists and internal medicine, was conducted. The pancreatic mass with liver metastases was deemed likely pancreatic adenocarcinoma. Given the patient’s poor baseline function and advanced disease, the MDT recommended supportive care and expedited discharge. The patient remained in hospital for 30 days while investigations were conducted until a palliative care plan was established, enabling fast-track discharge home, where the patient passed away three weeks later.

## Discussion

The pathophysiology of ODS is attributed to rapid osmotic shifts that damage the myelin sheaths in the central nervous system, and the rapid correction of sodium is the most commonly attributed trigger. Our case demonstrated a cancer-complicated ODS without hyponatraemia or rapid sodium correction. We believe this to be the first case report of ODS linked to pancreatic cancer with normal sodium levels.

There is a growing recognition that malignancy itself can contribute to the development of ODS. Although the exact mechanism is unknown, it has been hypothesised that nutrient deficiency plays a role in increasing the susceptibility of cancer patients to ODS. Ashrafian et al. proposed that oligodendrocytes depend on Na-K-ATPase for osmotic balance, and their vulnerability to energy depletion may contribute to ODS. Cancer patients have nutrient deficiencies which result in oligodendrocyte energy deficits and thus ODS [4]. Wang et al., in a scoping review, showed that 90.9% of ODS patients with cancer have other predisposing factors like malnutrition, alcohol intake and infection, which our patient appeared to have as well [3].

Although sodium levels remained within range, the potassium was low on admission and remained below range despite intravenous potassium fluids. Our patient was not aggressively resuscitated with fluids, which, however, appeared to be the trigger in a similar case of a patient with ODS and hypokalaemia reported by Ormonde et al. [5]. Low potassium level alone isn't believed to be a trigger by itself, as it cannot produce a significant shift in effective osmolality. This was also reported by Chung et al., in a case report on cholangiocarcinoma with hypokalaemia and cisplatin chemotherapy [6], where chemotherapy agents themselves were identified as a trigger of ODS [3].

The consistently elevated glucose levels in our patient without evidence of a hyperosmolar hyperglycaemic state (HHS) have been previously reported as a trigger for ODS. Mir et al. and Talluri et al. reported cases of ODS triggered by longstanding uncontrolled type 2 diabetes with hyperglycemia and normal sodium [7,8]. Donnelly et al. reported a similar case but with type 1 diabetes [9]. The clinical outcome of these cases varies with some even showing complete resolution. This underpins the variation ODS can have and how multiple factors can play a role in determining the prognosis. 

Hypoalbuminaemia, observed in our patient, is common in advanced malignancies and results from reduced hepatic synthesis and systemic inflammation. Albumin maintains oncotic pressure, and its depletion leads to fluid extravasation, contributing to the patient’s bilateral pitting pedal oedema. Nutritional deficiencies, common in pancreatic adenocarcinoma due to metabolic alterations and reduced intake, likely exacerbated this. Portal hypertension from liver metastases, which was observed in this case, and lymphatic obstruction further compounded the oedema [10-12]. Hypoalbuminaemia reflects a multifactorial process that is difficult to reverse without addressing the underlying malignancy, highlighting the need for supportive measures such as nutritional optimisation. Similar cases linking hypoalbuminaemia to ODS have been reported in diffuse large B-cell lymphoma by Nguyen et al., Mareli et al. and Kawata et al. [13-15].

The treatment of ODS remains a topic of debate, with no established consensus. Dopamine agonists and other anti-dystonic medications have shown promise in some cases involving extrapyramidal features [16]. Reinduction of hyponatremia using desmopressin, intravenous 5% dextrose solution, or both has been proposed for patients at low to moderate risk of ODS. Other therapeutic approaches, including corticosteroids, minocycline, plasmapheresis, and intravenous immunoglobulin, have also been explored [17]. In our patient, treatment with intravenous steroids led to a marked improvement in cognition, with the patient regaining the ability to speak in short sentences and showing reduced confusion. Although the patient demonstrated some upper extremity movement, they remained bedbound.

It is worth mentioning that the presentation of encephalitis following pancreatic cancer with liver metastasis initially raised the suspicion of paraneoplastic limbic encephalitis (LE). LE is an autoimmune condition linked to cancer, where antibodies target intracellular antigens giving rise to inflammation and injury to the limbic structures. Certain types of cancer, such as small cell lung cancer (SCLC), are more commonly associated with paraneoplastic LE. LE can be diagnosed through antibody blood tests targeting specific monoclonal antibodies such as anti-Hu, anti-Yo, anti-Ri, anti-amphiphysin, and anti-Ma2. MRI imaging often demonstrates abnormalities in the temporal lobe or EEG findings indicating epileptic activities localised in the temporal lobe [18]. In our patient, antibody testing was negative for these markers, and while a lumbar puncture was not performed, MRI of the brain demonstrated osmotic demyelination instead. These results collectively made the diagnosis of LE less likely.

## Conclusions

In this case, we presented a 52-year-old male with advanced pancreatic adenocarcinoma and metastatic liver disease who developed osmotic demyelination syndrome (ODS) despite normal sodium levels. ODS is a rare disorder traditionally associated with rapid correction of hyponatraemia, but our patient demonstrated multiple contributing factors, including malignancy, malnutrition, and hyperglycaemia. These factors, as highlighted in the literature, impair oligodendrocyte energy metabolism and disrupt osmotic homeostasis, predisposing patients to ODS.

While no single trigger could be identified in this case, it underscores the multifactorial nature of ODS and its potential occurrence even in the absence of hyponatraemia. Our findings contribute to the growing body of evidence linking malignancy and metabolic disturbances to ODS and highlight the need for further research to elucidate the precise pathophysiological mechanisms and improve management strategies for this complex condition.
